# The Effects of *Allium sativum* L., *Artemisia absinthium* L., *Cucurbita pepo* L., *Coriandrum sativum* L., *Satureja hortensis* L. and *Calendula officinalis* L. on the Embryogenesis of *Ascaris suum* Eggs during an In Vitro Experimental Study

**DOI:** 10.3390/pathogens11091065

**Published:** 2022-09-19

**Authors:** Mihai-Horia Băieş, Călin Gherman, Zsolt Boros, Diana Olah, Ana-Maria Vlase, Anamaria Cozma-Petruț, Adriana Györke, Doina Miere, Laurian Vlase, Gianina Crișan, Marina Spînu, Vasile Cozma

**Affiliations:** 1 Department of Parasitology and Parasitic Disease, Faculty of Veterinary Medicine, University of Agricultural Sciences and Veterinary Medicine of Cluj-Napoca, 3–5 Mǎnǎştur Street, 400372 Cluj-Napoca, Romania; 2 Department of Infectious Diseases, Faculty of Veterinary Medicine, University of Agricultural Sciences and Veterinary Medicine of Cluj-Napoca, 3–5 Mǎnǎştur Street, 400372 Cluj-Napoca, Romania; 3 Department of Pharmaceutical Botany, Faculty of Pharmacy, “Iuliu Haţieganu” University of Medicine and Pharmacy, 23 Gheorghe Marinescu Street, 400337 Cluj-Napoca, Romania; 4 Department of Bromatology, Hygiene, Nutrition, Faculty of Pharmacy, “Iuliu Haţieganu” University of Medicine and Pharmacy, 6 Pasteur Street, 400349 Cluj-Napoca, Romania; 5 Department of Pharmaceutical Technology and Biopharmaceutics, Faculty of Pharmacy, “Iuliu Haţieganu” University of Medicine and Pharmacy, 12 Ion Creangǎ Street, 400010 Cluj-Napoca, Romania; 6 Academy of Agricultural and Forestry Sciences Gheorghe Ionescu-Siseşti (A.S.A.S), 61 Mǎrǎşti Boulevard, 011464 Bucharest, Romania

**Keywords:** *Ascaris suum*, Romanian medicinal plants, antihelminthics

## Abstract

*Ascaris suum* is present in traditionally managed indoor pig herds and in industrialized farms, especially in older fatteners and sows. The increasing resistance to common antihelminthic drugs redirected research towards alternative and traditional therapies, which also include medicinal plants. This study comparatively evaluated the in vitro antiparasitic effects of *Allium sativum* L., *Artemisia absinthium* L., *Cucurbita pepo* L., *Coriandrum sativum* L., *Satureja hortensis* L. and *Calendula officinalis* L. against *A. suum* egg hatching and larval development. *A. suum* eggs were sampled from randomized fecal specimens collected from traditionally raised swine. The egg suspension (ES, 12 × 10^3^/mL) was divided into two controls (C) (1C—1 mL ES + 1 mL distilled water, 2C—five plates of 1 mL ES + 1 mL ethanol of 70%, 35%, 17.5%, 8.75%, and 4.375%, respectively) and six experimental groups, and placed in 3 mL cell plates. The experimental groups (EG, 1–6) included ES + each alcoholic plant extract (10%, 5%, 2.5%, 1.25%, 0.625%). Both C and EG were performed in quintuplicate. All variants were incubated at 27 °C for a total of 21 days. *A. suum* eggs were examined after 2, 14 (L1), and 21 (L2/L3) days of incubation. The efficacy of all tested plant extracts increased with concentration. Anti-embryogenic effects on *A. suum* eggs were expressed by all plants. A superior influence was observed in *A. sativum* L., *A. absinthium* L., *C. pepo* L. and *S. hortensis* L. extracts, at all concentrations tested. *A. sativum* L. and *A. absinthium* L. extracts showed the strongest antihelminthic activity, while *C. sativum* L. and *C. officinalis* L. were the weakest ascaricids. Future in-depth phytochemical studies are required to identify the compounds responsible for the anthelminthic properties of these plant species.

## 1. Introduction

Nowadays, studies regarding the effects of various plant extracts on pathogenic microorganisms are intensively developing. Due to huge plant biodiversity in the world, it is important to discover the effects of plant extracts and specific compounds from some plant species with antioxidant and antimicrobial potential [1,2,3,4,5].

*Ascaris suum* is an intestinal parasite in pigs, with a worldwide distribution. The infestation can have low to moderate pathogenic effects on the health and productivity of animals. In addition to these direct effects, there may be other indirect effects leading to increased host susceptibility to bacterial or viral infections, related to the migratory and immunomodulatory capacity of the parasite [6]. 

*Ascaris lumbricoides* is a soil-borne nematode which can cause human infestation by ingestion of food contaminated with embryonated eggs. For several reasons, recent studies on nematodes, especially on the genus *Ascaris*, have regarded *A. suum* infection in pigs as a model for human infection with *A. lumbricoides*. *A. lumbricoides* and *A. suum* are closely related roundworm species and both exhibit natural host-parasite relationships, having similar biological cycles [7]. *Ascaris* eggs have the highest resistance and survive under numerous treatment conditions, such as a variety of strong acids, strong alcali, oxidants, reductants, protein disruptors, and surfactants. Several studies have been performed to inactivate *A. suum* larvae using fatty acids, high hydrostatic pressure, low pressure, UV radiation and ammonia [8].

Lately, due to amplified resistance to classical antihelminthic drugs, the emphasis has shifted on the study of medicinal plants. These herbs have long been used to control parasitic diseases and, in many parts of the world, they are still applied for this purpose. In ethno-veterinary medicine, which draws inspiration from traditional practices, there seems to be a range of plants or plant extracts suitable for treating almost every parasitic disease of livestock [9]. Different in vitro methods, such as egg hatch and larval motility tests, are frequently implied in examining the effects of medicinal plants against nematodes [10].

The present study evaluated the anthelmintic activity of *Allium sativum* L., *Artemisia absinthium* L., *Coriandrum sativum* L., *Cucurbita pepo* L., *Satureja hortensis* L. and *Calendula officinalis* L. alcoholic extracts. These plants have been traditionally used as empirical therapy on the account of their antiparasitic effect. Medicinal plant extracts and their constituents exhibit various biological activities including anticarcinogenic, antioxidant, antidiabetic, renoprotective, cardioprotective, gastroprotective, neuroprotective, immunomodulatory, anti-inflammatory, antibacterial, antifungal, antiprotozoal and anthelmintic activities. The anthelmintic effect has been reported to be expressed by several plant-derived bioactive compounds, including polyphenols, methoxylated flavones, allicin, and cucurbitine [3,10,11,12,13,14,15,16,17,18,19,20].

To our best knowledge, there are no reports on the anthelmintic activity of Romanian medicinal plants in swine. Therefore, the current study aimed at evaluating the in vitro effect of six ethanolic medicinal plant extracts on the embryogenic development of *A. suum* eggs.

## 2. Results

### 2.1. Analysis of Plant Extracts

The major compounds identified following the chemical analysis of the 10% alcoholic plants extracts were as follows: for *A. sativum* L. (poliphenols, tocopherols, sulfoxide), for *A. absinthium* L. (poliphenols, tocopherols, sterols, methoxylated flavones, sesquiterpene lactones), for *C. sativum* L. (poliphenols, sterols), for *C. pepo* L. (tocopherols, sterols), for *S. hortensis* L. (poliphenols, tocopherols, sterols, methoxylated flavones) and for *C. officinalis* L. (poliphenols, tocopherols, sterols). These compounds are presented in detail in Table 1.

### 2.2. Analysis of Plant Extracts Activity 

**Figure 1:**
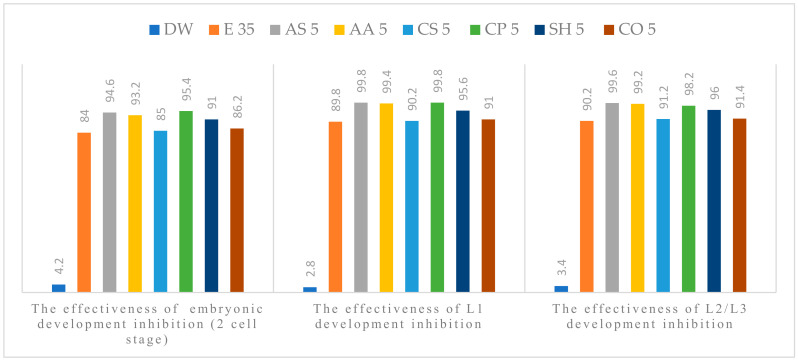
Percentage of embryogenesis inhibition at 5 % concentration: Distilled water (DW), Ethanol (E), *A. sativum* L. (AS), *A. absinthium* L. (AA), *C. sativum* L. (CS), *C. pepo* L. (CP), *S. hortensis* L. (SH), *C. officinalis* L. (CO).

**Figure 2:**
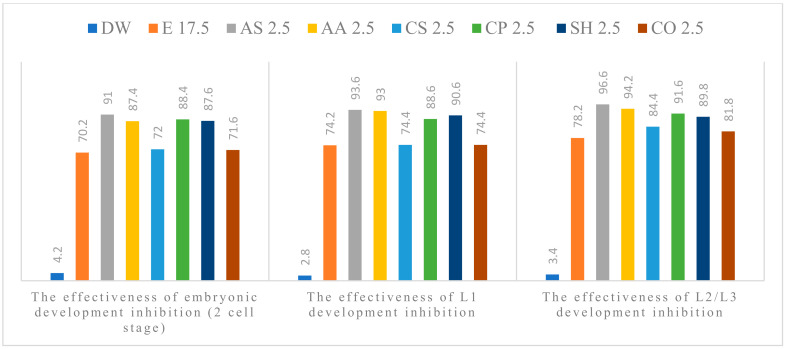
Percentage of embryogenesis inhibition at 2.5% concentration: Distilled water (DW), Ethanol (E), *A. sativum* L. (AS), *A. absinthium* L. (AA), *C. sativum* L. (CS), *C. pepo* L. (CP), *S. hortensis* L. (SH), *C. officinalis* L. (CO).

**Figure 3:**
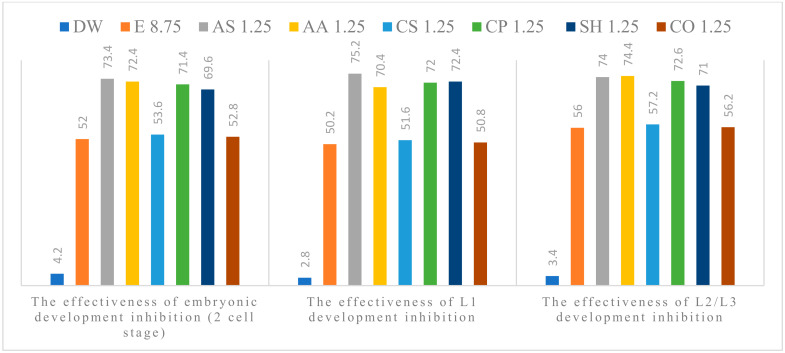
Percentage of embryogenesis inhibition at 1.25%concentration: Distilled water (DW), Ethanol (E), *A. sativum* L. (AS), *A. absinthium* L. (AA), *C. sativum* L. (CS), *C. pepo* L. (CP), *S. hortensis* L. (SH), *C. officinalis* L. (CO).

**Figure 4:**
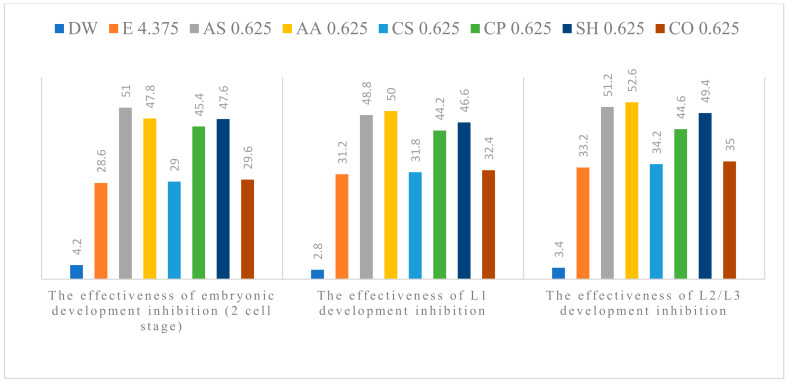
Percentage of embryogenesis inhibition at the concentration of 0.625%: Distilled water (DW), Ethanol (E), *A. sativum* L. (AS), *A. absinthium* L. (AA), *C. sativum* L. (CS), *C. pepo* L. (CP), *S. hortensis* L. (SH), *C. officinalis* L. (CO).

**Figure 5:**
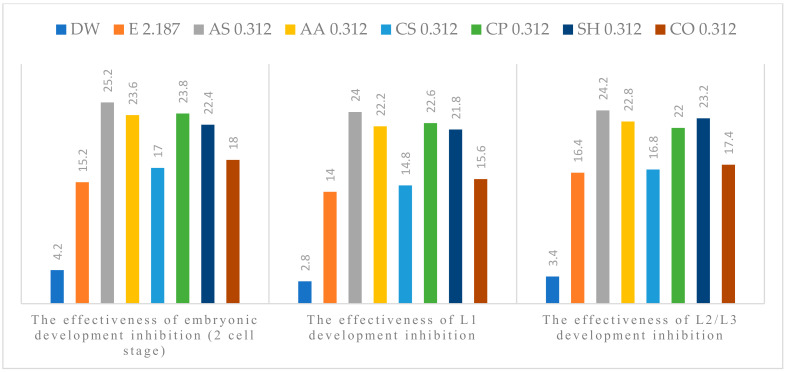
Percentage of embryogenesis inhibition at the concentration of 0.312%: Distilled water (DW), Ethanol (E), *A. sativum* L. (AS), *A. absinthium* L. (AA), *C. sativum* L. (CS), *C. pepo* L. (CP), *S. hortensis* L. (SH), *C. officinalis* L. (CO).

The anthelmintic efficacy in the experimental groups increased with concentration. All tested plant extracts showed anti-parasitic effect on the development of *A. suum* eggs, at all concentrations used. Alcoholic plant extracts (APE) of *A. sativum* L., *A. absinthium* L., *C. pepo* L. and *S. hortensis* L. were the most effective (Figure 1, Figure 2, Figure 3, Figure 4 and Figure 5). The effect of APE escalated in proportion along with the increasing concentration. The real effectiveness of APE in embryonic development inhibition was between 0.4–22.4%: for L1 development inhibition ranged between 0.2–25% and for L2/L3 development inhibition ranged between 0.2–19.4%, respectively. These results depended on the concentration used (Table 2).

## 3. Discussion

Recently, there has been a growing awareness of the risks posed to human health by drug residues in food. Furthermore, the augmentation of anthelmintic resistance in animals has led to increased interest in the use of herbs and metabolites as an anthelmintic organic alternative [21]. Development of anthelmintic resistance is an inevitable consequence of irrational anthelmintic treatments [22]. Numerous parasites of veterinary importance have genetic features that favor development of anthelmintic resistance [23]. Similarly, it has been observed that frequent usage of the same class of anthelmintic, sub-optimal dosage, prophylactic mass treatment of animals, frequency of treatments, lack of coproparasitological examination before therapy have contributed to the widespread development of anthelmintic resistance [22,23]. Therefore, the introduction of new drug classes is necessary. Furthermore, parasite control programs must be designed towards enhanced sustainability and lesser aggressiveness, i.e., including parasite resistant breeds of livestock, antiparasitic vaccines, tailored nutrition, pasture management, nematode-trapping fungi and botanical dewormers. Testing for anthelmintic resistance needs to be a routine component of livestock preventive health programs [22,23].

*A. sativum* L. bulbs carry hundreds of phytochemicals, including sulfur-containing compounds such as ajoene, thiosulfinate (allicin), vinyldithiine, sulfide (diallyl disulfide and trisulphide) and others, which account for 82% of the total sulfur content of garlic [6]. The alcoholic extract from the bulb of *A. sativum* L. demonstrated moderate in vitro anti-parasitic behavior against *A. lumbricoides* [21]. In a study by Dibfiora et al. (2021) [24], the effectiveness of *Allium cepa* L. and *A. sativum* L. extracts on ascarids was also demonstrated in vitro. Another study reported in vitro antihelminthic activity of aqueous and ethanolic extract of *A. cepa* and *A. sativum* bulbs on *Ancilostoma caninum* and *Toxocara canis* starting from the minimum concentration of 0.61 µg/mL [25]. The antiparasitic effect of garlic infusion was also shown in previously published studies. The infusion killed all the adult stages of *Ascaridia galli*, and the higher the concentration of the extract, the faster the adult parasites were destroyed [21,26]. Moreover, the aqueous and alcoholic extracts of *Allium sativum* L. proved a good anthelmintic effect on *Capilaria* spp. in fish and sheep [27,28]. In the present study, the ethanolic garlic extract was the most effective extract at all concentrations used. This effectiveness is mainly due to sulfoxide and polyphenols, defence molecules which are present in a fairly high concentration in this plant. Although it failed to destroy *A. suum* eggs, the solution led to the inhibition of embryogenesis and, in some cases, to the inhibition of larval mobility.

The active constituents present in *A. absinthium* L. are the bitter substances (sesquiterpene lactones), essential oils (myrcene, sabinene, linalool and trans-sabinyl acetate) and other compounds (flavonoid, sterolic and polyphenolic compounds, phenolic acids, lignans, sequiterpenes) [13,29]. Urban et al. (2008) [10] have shown the antiparasitic effect of ethanolic extract on the embryogenesis inhibition of *A. suum* eggs. In another study, a reduction of *Toxocara* eggs was observed in cats receiving *A. absinthium* L. orally, but in vitro, the *A. absinthium* L. extract did not inhibit larval development [30]. *Artemisia* species are known to have antiparasitic efficacy on *Hemonchus* eggs, which decreased in number in sheep feces after administration of *A. absinthium* L. extract. The extract effect is expressed by destroying the parasite or causing its paralysis. As for the in vitro exposure, it diminishes the mobility of the larvae [18,31]. The survival rate of *Trichinella spiralis* larvae was reduced in the muscles of rats after 20 consecutive days of 300 and 600 mg/kg doses of *A. absinthium* L. extract. In the in vitro protocols, the essential oil proved a lethal effect on the larvae [18,32]. In the present study, wormwood ethanolic extract was the second most effective extracts at all concentrations used. The anthelmintic effect may be due to the polyphenols that are found in abundance in this plant (Table 1). Although the mode of anti-parasitic action of these active principles is poorly understood, the effectiveness of embryogenesis inhibition is high. 

Coriander (*C. sativum* L.) is an aromatic and medicinal plant that significantly improves health of animals and humans. Coriander contains essential oils, flavonoids, fatty acids, isocoumarin sterols, and phenolic compounds [15,33,34]. Coriander seeds contain about 1% essential oil, and linalool is the main active component [12]. The antiparasitic efficacy of *C. sativum* L. essential oils/aqueous and alcoholic extracts was studied mainly in sheep nematodes by two in vitro tests using the egg hatch test (EHT) and the larval development test (LDT) [35,36,37]. *C. sativum* L. essential oils had a dose-dependent effect in EHT, inhibiting 81.2% (hatching) of *H. contortus* larvae at a concentration of 2.5 mg/mL. In LDT, *C. sativum* L. essential oil, at a concentration of 10–20 mg/mL, inhibited over 93% of the development of digestive strongyle larvae [35,38]. Hosseinzadeh et al. (2016) [39] compared the in vitro antiparasitic efficacy of niclosamide with the alcoholic extract of *C. sativum* L. on adults of *Hymenolepis nana* and found that the addition of 1000 mg/mL niclosamide paralyzed and destroyed the worms in 5 min, while *C. sativum* L. destroyed them in 30 min. Boros et al. (2021) [40] demonstrated the efficacy of *C. sativum* L. ethanolic extract on *T. britovi* and *T. spiralis* larvae, which at concentrations of 2.5, 5, and 10% led to complete inhibition of larval mobility. In the present study, the coriander extract had the lowest efficacy in egg hatch inhibition/larval development inhibition, results comparable to the control group treated with ethanol. Although this extract is quite rich in polyphenols, their concentration is low compared to that in wormwood. The anthelmintic effect was very weak, possibly due to the thick shell of the *A. suum* eggs. 

The antihelminthic (ovicidal and larvicidal) activity of *C. pepo* L. seeds has been linked to the active phytochemical components, including alkaloids, flavonoids, tannins, saponins, terpenoid phenolic compounds and glycosides [41]. Aziz et al. (2018) [42] showed the in vitro lethal effect of pumpkin seed ethanolic extract against adults of *A. galli* at concentrations of 25, 50, and 75 mg/mL, an effect comparable to the group subjected to the action of fenbendazole. *A. galli* eggs treated with pumpkin seed extract concentrations of 5–20 mg/mL led to eggs/larval development inhibition, results comparable to the group treated with levamisole [41]. Pumpkin seed extract also showed a strong in vitro antihelminthic effect against *A. suum* adults. Thus, at a concentration of 54.5%, adult worms’ death occurred in 11 h and 48 min, while at a concentration of 70.5%, in 7 h and 48 min [43]. In a study which tested the effectiveness of pumpkin oil on eggs/larvae of *T. cati*, the oil led to pronounced egg development inhibition, respectively to the death of larvae in a fairly high percentage [19]. The inhibitory antiparasitic effect on *A. suum* eggs’ hatching and larval mobility was also demonstrated at concentrations between 62.5–2000 µg/mL [10]. In our study, *C. pepo* ethanolic extract proved to be very effective (ranked third) in egg development inhibition at all tested concentrations. Responsible for the anthelmintic effectiveness is most likely to be cucurbitine [19], a natural compound contained by plants of the *Cucurbita* genus. 

The main bioactive substances in the summer savory plant (*S. hortensis* L.) are thymol, carvacrol, phenols and flavonoids [11,44]. Carvacrol and γ-terpene are major components of *S. hortensis* L. essential oil, which at concentrations of 1 and 2 mg/mL showed a 100% scolicidal power (hydatic chyst) after 20 min of exposure [45]. Urban et al. (2014) [46] demonstrated the in vitro efficacy of the ethanolic extract of *S. hortensis* L. against adults of *Chabertia ovina*, which at concentrations of 0.25, 0.5, 1 and 2 mg/mL led to the inhibition of their motility in a quite high and concentration dependent percentage. Urban et al. (2008) [10] studied the anthelmintic activity of the ethanolic extract of *S. hortensis* L. against *A. suum* eggs and *Trichostrongilus colubriformis* infectious larvae. According to the report, the extract did not show strong activity against these two nematodes. *S. hortensis* L. essential oil showed the strongest ovocidal activity (99.3–100%) against gastrointestinal nematodes in sheep, at all concentrations tested [47,48]. The hydro-alcoholic extract of *S. hortensis* L. had a poor efficacy in egg hatching inhibition, respectively in larval mobility inhibition of equine strongylidosis [49]. In our study, the plant extract showed a strong efficacy in egg/larval development inhibition with all tested concentrations, effect comparable to garlic, wormwood, and pumpkin extracts. We assume that the very strong anthelmintic effect of this plant is due to its high content in polyphenols and methoxylated flavones. 

Marigold (*C. officinalis* L.) has various biologically active constituents, such as carotenoids, flavonoids, saponins, sterols, phenolic acids, volatile oils, coumarins. It has been reported that various parts of the plant, such as the leaves and flowers, possess therapeutic activity [50]. Several pharmacological studies have reported that *C. officinalis* L. has a wide range of properties, such as anti-inflammatory, antibacterial, antifungal, antiparasitic against giardiasis, trichomoniasis, leishmaniasis etc., antiviral and antitumoral [15,16,37]. Although there are no current studies on the anti-parasitic effect of this plant in pigs, there is research on the activity of essential oils and aqueous extract of *C. officinalis* L., revealing that they had no effect on the embryogenesis of *A. suum* eggs, but did have a 50% efficacy on L1-2 larvae of *Strongiloides papillosus* [51]. Certain bioactive principles (oleane-type glucuronides) isolated from *C. officinalis* L. seem to affect the development of the free-living stages of *Heligmosomoides bakeri*, a mouse nematode [52]. In the present study, the marigold extract had the lowest efficacy in egg hatch inhibition/larval development inhibition, results comparable to the control and experimental groups treated with ethanol and coriander alcoholic extract, respectively. To date, the effect of this plant on helminths has been hardly studied. Still, it is known to have an antiparasitic effect on protozoa.

The chemical composition of the six Romanian medicinal plants used in the present study is similar to that reported in the specialized literature. The differences between the main biological compounds are given by their concentration [11,12,13,15,16,17,18,29,37].

In summary, based on the results of the above-described experiments and the specialised literature, the plant extracts used in our experiment could be prospective sources for the development of new potent anthelmintic herbal remedies both for humans and animals [10,13,17,19].

The alcoholic plants extracts can be used in vivo, after removing the ethanol, without any side effects if the correct dosage is implemented [42,53]. Based on our results, the ethanolic extracts of *A. sativum* L., *A. absinthium* L., *C. pepo* L., and *S. hortensis* L. can be administered for anthelmintic treatment, while the ethanolic extracts of *C. sativum* L. and *C. officinalis* L. may be used only to control the population of parasites.

The findings of the present study will hopefully contribute to the field of plant antihelminthic preparations to develop sustainable, effective, and safe alternatives to conventional antihelminthic drugs. The antihelminthic activity exhibited by some APE extracts against *A. suum* eggs in this study bears prominent importance considering the worldwide emergence of anthelmintic resistance. Further studies on standardization of doses, determining an efficient herbal formula and identifying the mechanism of action of these extracts against *A. suum* are regarded as immediate steps of the overall research. The use of herbal remedies as antihelminthics based on local plants will at least offer a cheap, reliable and a readily available alternative to highly expensive and unavailable conventional anthelmintics to the struggling low-income farmers worldwide.

## 4. Materials and Methods

### 4.1. Preparation of A. suum Eggs Stock Solution

Adult *A. suum* parasites were obtained from the proximal portion of the small intestine of four pigs from low-input farms, traditionally slaughtered for consumption. The females were separated from males, and the latter were eliminated. Female *A. suum* (n = 22) were identified, separated, and rigorously washed with saline water. The uteri were isolated and both anterior portions were dissected under magnification (4×). Eggs were recovered from the uteri using an automatic pipette and suspended in warm distilled water in Petri dishes. Finally, a suspension of non-embryonated eggs (12,000 eggs/mL) was obtained. The exposure of eggs to alcoholic plant extracts occurred in the same day [54,55,56].

### 4.2. Obtainment of Alcoholic Plant Extracts

The alcoholic plant extracts were obtained as follows: 10 g of each plant were let to macerate for 72 h in 100 mL of 70% ethanol, after which they were transferred to a turbo extractor where they were extracted for 3 min at 4000 rpm, and the extract was finally filtered. We used the aerial parts of wormwood, marigold and summer savory, coriander and pumpkin seeds, and garlic bulbs, respectively. Six plant extracts (*A. sativum* L., *A. absinthium* L., *C. sativum* L., *C. pepo* L., *C. officinalis* L. and *S. hortensis* L.), which had an initial concentration of 10%, were hence obtained. The plant extracts were obtained at the “Iuliu Haţieganu” University of Medicine and Pharmacy Cluj-Napoca, where the chemical composition of each extract was also performed [57,58,59,60,61,62,63,64,65,66]. High performance liquid chromatography coupled with mass spectrometry (LC/MS) was employed for the analysis of major compounds present in the plant extracts (Table 2). The experiment was performed by using an Agilent 1100 HPLC Series system (Agilent Technologies, Santa Clara, CA, USA) equipped with binary pump, degasser, column thermostat, autosampler, and UV detector. The HPLC system was coupled with a mass spectrometer, type Brucker Ion Trap SL (Brucker Daltonics GmbH, Leipzig, Germany). For the separation, a reverse-phase analytical column was used (Zorbax SB-C18 100 × 3.0 mm i.d., 3.5 μm particle). Depending on the chemical class of each compound from the plant samples, different ionisation sources and mode were employed for the MS system. More precisely, for analysis of alliin, methoxylated flavones, and polyphenols, the ESI (electrospray ionisation) source was used, whereas APCI (atmospheric pressure chemical ionization) source was employed for analysis of sesquiterpene lactones, sterols, and tocopherols. The chromatographic data were processed by using ChemStation and DataAnalysis software from Agilent (Agilent Technologies, Santa Clara, CA, USA).

### 4.3. Experimental Design 

Two control groups (ethanol and distilled water) and six experimental groups (*A. sativum* L., *A. absinthium* L., *C. sativum* L., *C. pepo* L., *S. hortensis* L. and *C. officinalis* L.) were established. Initially, the concentration of the ethanolic extracts of wormwood, garlic, pumpkin, coriander, summer savory and marigold was of 10%. There were 5 successive dilutions (variants) of each alcoholic extract (10%, 5%, 2.5%, 1.25%, 0.625%), as well as for ethanol, thus five replicates per experimental variant were made. The final concentrations used for each extract were of 50, 25, 12.5, 6.25, 3.12 mg/mL. ELISA plates with 24 wells were incubated at 27 °C degrees for 21 days. The samples were individually stirred for oxygenation, daily, and a light schedule of 10 h per day was established, thereby simulating the light/dark alternation (Table 3) [55,67].

### 4.4. Eggs Hatch Test/Larval Development Assay

**Figure 6:**
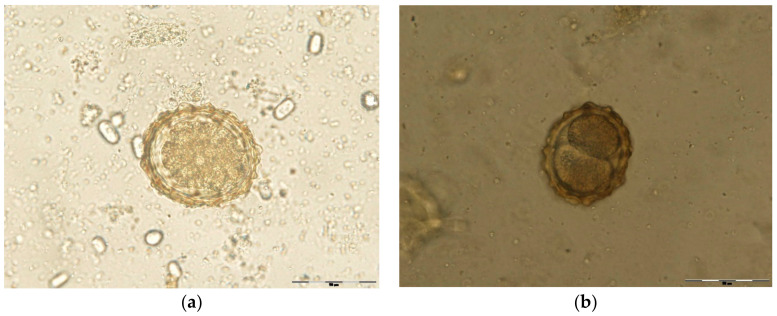
(**a**) *Ascaris suum* unembryonated egg (400×); (**b**) Egg of *A. suum* in two stage cell (early morula, 400×).

**Figure 7:**
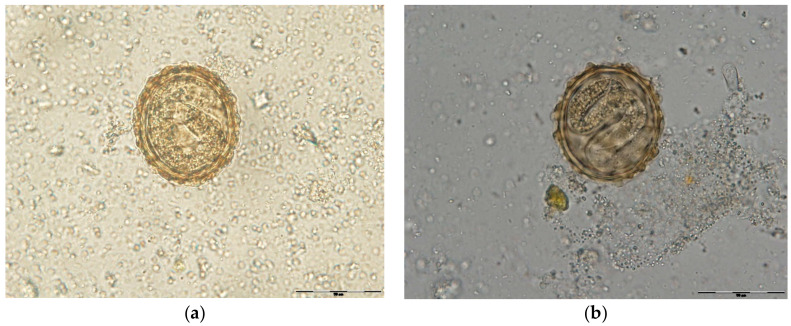
(**a**) *A. suum* egg with L1 (larva, 400×); (**b**) *A. suum* egg with L 2/3 (larva, 400×).

**Figure 8:**
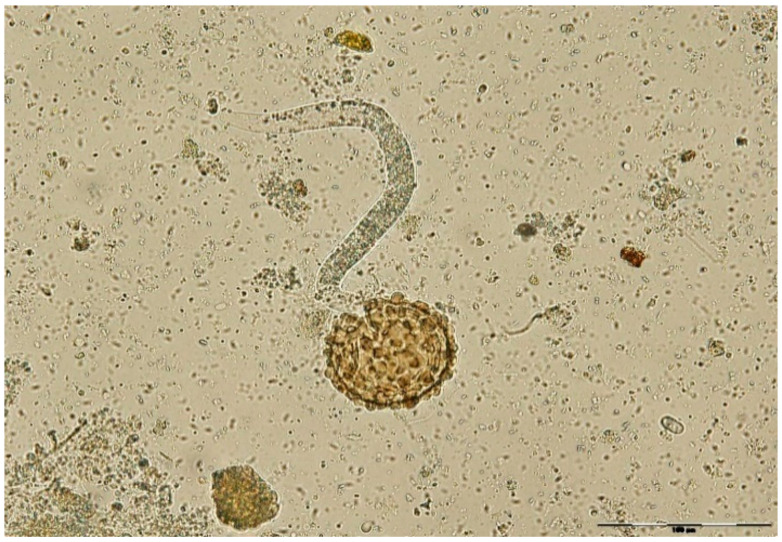
Hatched L 2/3 (larva, 400×).

To this purpose, both control groups and experimental groups were incubated at 27 °C for 48 h. Unembrionated and two stage cells eggs were counted, while destroyed eggs were not included in the study (Figure 6). The egg hatch inhibition test was performed using alcoholic plant extracts at different concentrations. On day 2, the efficacy of two stage cells development inhibition (early morula) was calculated. Incubation continued until day 21 in order to test the potential larvicidal effect of alcoholic plant extracts (Figure 7 and Figure 8). On day 14 and day 21, the effectiveness of L1, respectively L2/L3 development inhibition was calculated. Every group was replicated five times and 100 eggs per dilution were examined (500 eggs/dilution). 

The percentage inhibition of egg hatching/larval development was calculated using the following formula **(A − B)/A × 100** [25]: where A stands for the estimated total number of eggs and B for the number of hatched or developing eggs with larvae. 

Finally, the real therapeutic efficacy of plant extracts in inhibiting embryogenesis was determined with the following formula: **(A − B) − (C − B)**, where A = experimental groups; B = groups treated with distilled water; C = groups treated with ethanol, at all above mentioned concentrations. 

### 4.5. Statistical Analysis and Ontologies/Pathogens, Diseases, Medicinal Plants and Chemical Compounds

First, the mean and standard deviation of the mean were calculated for the inhibition of early morula (two stage cell) and inhibition of larval development (L1 larva on day 14 and L2/3 larva on day 21). The ANOVA program was then used to compare the groups tested with extracts of garlic, wormwood, pumpkin, coriander, summer savory and marigold with the control groups, and the experimental groups were compared with each other. A value of *p* ≤ 0.05 was considered statistically significant. Statistical analysis was performed using Excel and ANOVA. All processed data are detailed in the Tables S1–S3. The ontologies/pathogens, diseases, medicinal plants, and chemical compounds have been described in Table S4.

## 5. Conclusions

In the current study, all plants extracts showed various degrees of inhibitory effects on egg development. *A. sativum* L., *A. absinthium* L., *C. pepo* L. and *S. hortensis* L. extracts have shown the strongest anthelmintic activity. In summary, based on the results of this study we suggested that some of these plant materials could be prospective sources for development of new antiparasitic herbal remedies. Given that these plants are commonly found in the native flora of Romania, they could be easily introduced into animal feed. However, in-depth phytochemical studies are needed to identify the compounds, doses responsible for the anthelmintic properties of these species. To our knowledge, this is the first ethno-pharmacological report based on the anthelmintic activity of medicinal plants traditionally used to treat *A. suum* infection in Romania.

## Figures and Tables

**Table 1:** The chemical composition of alcoholic plant extracts (10%) using LC/MS.

Bioactive Compounds		Vegetal Species and Plant Part Used for Extraction and LC-MS Analysis					
		*Artemisia absinthium* L.	*Satureja hortensis* L.	*Calendula officinalis* L.	*Allium sativum* L.	*Coriandrum sativum* L.	*Cucurbita pepo* L.
		herba	herba	herba	bulbus	fructus	semen
**Polyphenols (μg/mL)**	Chlorogenic acid	107.15	<LOQ	220.767	-	4.177	-
	Caffeic acid	-	<LOQ	-	1.221	-	-
	*p*-coumaric acid	0.621	1.464	-	-	0.501	-
	Ferulic acid	0.759	0.557	-	0.456	0.759	-
	Sinapic acid	-	-	-	0.228	-	-
	Vitexin	1.631	-	-	-	-	-
	Isoquercitrin	56.754	6.515	38.877	-	-	-
	Rutoside	3.826	<LOQ	18.819	-	<LOQ	-
	Quercitrin	1.113	0.365	<LOQ	-	-	-
	Quercetol	6.285	0.394	-	-	-	-
	Luteolin	1.159	6.621	-	-	-	-
	Kaempferol	3.666	-	-	-	-	-
	Apigenin	0.481	2.442	-	-	-	-
	Syringic acid	1.85	2.28	1.51	-	0.09	-
	Protocatechuic acid	1.32	0.95	0.67	-	-	-
	Vanillic acid	1.98	0.65	0.44	-	0.94	-
**Tocopherols (ng/mL)**	α-tocopherol	50.0	86.8	61.6	36.1	-	-
	γ-tocopherol	23.8	89.0	248.9	-	-	446.0
	Δ-tocopherol	5.0	13.2	9.3	-	-	23.2
**Sterols (μg/mL)**	Ergosterol	0.344	1.420	0.500	-	0.584	-
	Stigmasterol	34.831	14.215	72.888	-	9.675	22.024
	Β-sitosterol	140.985	313.315	241.997	-	31.548	5.355
	Campesterol	3.329	6.140	1.635	-	1.780	0.358
**Methoxylated flavones (ng/mL)**	Jaceosidin	-	8820.76	-	-	-	-
	Hispidulin	3047.92	2483.00	-	-	-	-
	Eupatorin	976.53	-	-	-	-	-
	Casticin	15,384.14	-	-	-	-	-
	Acacetin		12,691.97	-	-	-	-
**Sesquiterpene lactones (ng/mL)**	α-santonin	450.52	-	-	-	-	-
	Vulgarin	6499.39	-		-	-	-
**Sulfoxide (μg/mL)**	Aliin	-	-	-	14.726	-	-

LC/MS—high performance liquid chromatography coupled with mass spectrometry; “-”—Not found; <LOQ—identified based on MS spectra but not determined quantitatively, below limit of quantification.

**Table 2:** The real therapeutic efficacy (±SDM) of alcoholic plant extracts in embryogenesis inhibition.

	Real Effectiveness of Alcoholic Plant Extracts		
**Groups %**	**2 Days % (±SDM)**	**14 Days % (±SDM)**	**21 Days % (±SDM)**
**AS 5**	10.6 (±2.30)	10 (±1.58)	9.4 (±1.81)
**AS 2.5**	20.8 (±0.83)	19.4 (±4.61)	18.4 (±2.88)
**AS 1.25**	21.4 (±3.28)	25 (±3.08)	18 (±2)
**AS 0.625**	22.4 (±2.40)	17.6 (±3.04)	18 (±4.35)
**AS 0.312**	10 (±4.41)	10 (±2.34)	7.8 (±1.30)
**AA 5**	9.2 (±3.63)	9.6 (±1.81)	9 (±1.22)
**AA 2.5**	17.2 (±3.63)	18.8 (±1.48)	16 (±3.16)
**AA 1.25**	20.4 (±1.94)	20.2 (±5.01)	18.4 (±4.39)
**AA 0.625**	19.2 (±3.34)	18.8 (±3.63)	19.4 (±3.65)
**AA 0.312**	8.4 (±4.27)	8.2 (±3.11)	4.4 (±3.36)
**CS 5**	1 (±0.57)	0.4 (±0.28)	1 (±0.41)
**CS 2.5**	1.8 (±1.27)	0.2 (±0.14)	6.2 (±2.28)
**CS 1.25**	1.6 (±1.13)	1.4 (±0.98)	1.2 (±0.84)
**CS 0.625**	0.4 (±0.28)	0.6 (±0.32)	1 (±0.72)
**CS 0.312**	1.8 (±1.27)	0.8 (±0.56)	0.4 (±0.21)
**CP 5**	11.4 (±2.30)	10 (±1.58)	8 (±2.34)
**CP 2.5**	18.2 (±3.89)	14.4 (±2.70)	13.4 (±2.88)
**CP 1.25**	19.4 (±3.97)	21.8 (±1.79)	16.6 (±1.95)
**CP 0.625**	16.8 (±2.16)	13 (±3.39)	11.4 (±4.03)
**CP 0.312**	8.6 (±1.34)	8.6 (± 2.79)	5.6 (±2.07)
**SH 5**	7 (±1.87)	5.8 (±3.12	5.8 (±2.78)
**SH 2.5**	17.4 (±3.13)	16.4 (±2.60)	11.6 (±1.95)
**SH 1.25**	17.6 (±3.51)	22.2 (±4.54)	15 (±3.33)
**SH 0.625**	19 (±1.58)	15.4 (±1.51)	16.2 (±3.63)
**SH 0.312**	7.2 (±2.38)	7.8 (±2.48)	6.8 (±1.09)
**CO 5**	2.2 (±1.55)	1.2 (±0.73)	1.2 (±0.84)
**CO 2.5**	1.4 (±0.98)	0.2 (±0.09)	3.6 (±2.54)
**CO 1.25**	0.8 (±0.56)	0.6 (±0.42)	0.2 (±0.11)
**CO 0.625**	1 (±0.70)	1.2 (±0.66)	1.8 (±1.27)
**CO 0.312**	2.8 (±1.97)	1.6 (±1.13)	1 (±0.46)

*A. sativum* L. (AS), *A. absinthium* L. (AA), *C. sativum* L. (CS), *C. pepo* L. (CP), *S. hortensis* L. (SH), *C. officinalis* L. (CO), Standard deviation of the mean (SDM).

**Table 3:** Experimental design.

Control Groups			Experimental Groups	
Variants	E	DW	Variants	AS, AA, CS, CP, SH, CO
**E 35**	1 mL ES + 1 mL 70% E	1 mL ES + 1 mL DW	EG 5	1 mL ES + 1 mL 10% APE
**E 17.5**	1 mL ES + 1 mL 35% E		EG 2.5	1 mL ES + 1 mL 5% APE
**E 8.75**	1 mL ES + 1 mL 17.5% E		EG 1.25	1 mL ES + 1 mL 2.5% APE
**E 4.375**	1 mL ES + 1 mL 8.75% E		EG 0.625	1 mL ES + 1 mL 1.25% APE
**E 2.187**	1 mL ES + 1 mL 4.375% E		EG 0.312	1 mL ES + 1 mL 0.625% APE

ES—eggs suspension, EG—experimental group, APE—alcoholic plant extract, DW—distilled water, E—ethanol, AS—*A. sativum* L., AA—*A. absinthium* L., CS—*C. sativum* L., CP—*C. pepo* L., SH—*S. hortensis* L., CO—*C. officinalis* L.

## Data Availability

Not applicable.

## Supplementary Materials

The following supporting information can be downloaded at: https://www.mdpi.com/article/10.3390/pathogens11091065/s1, Table S1: The mean percentage (±SDM) for the control and experimental groups; Table S2: The mean percentage of embryogenesis inhibition (±SDM) from the controls and experimental groups at 2, 14 and 21 days. Table S3: The mean percentage of embryogenesis inhibition (±SDM) from the experimental groups at 2, 14 and 21 days. Table S4: Ontologies/pathogens, diseases, medicinal plants and chemical compounds used in the experiment.
