# Supplementary material for: The Effects of Allium sativum L., Artemisia absinthium L., Cucurbita pepo L., Coriandrum sativum L., Satureja hortensis L. and Calendula officinalis L. on the Embryogenesis of Ascaris suum Eggs during an In Vitro Experimental Study
Source: Pathogens. 2022 Sep 19;11(9):1065. doi: 10.3390/pathogens11091065 (PMC9501052; doi:10.3390/pathogens11091065)
Supplement: Supplementary file 1 [file pathogens-11-01065-s001.zip › pathogens-1890576-supplementary.pdf]

Supplementary material

Table S1. The mean percentage (±SDM) for the control and experimental groups**.**

Groups %	2 days	14 days	21 days
**DW**	4.2 (±1.92)	2.8 (±1.79)	3.4 (±2.7)
**E 35**	84 (±1.58)	89.8 (±1.92)	90.2 (±1.92)
**E 17.5**	70.2 (±2.39)	74.2 (±2.77)	78.2 (±1.48)
**E 8.75**	52 (±2.55)	50.2 (±3.11)	56 (±2.74)
**E 4 .35**	28.6 (±1.52)	31.2 (±1.92)	33.2 (±2.28)
**E 2.187**	15.2 (±1.92)	14 (±1.58)	16.4 (±1.52)
**AS 5**	94.6 (±2.07)	99.8 (±0.45)	99.6 (±0.89)
**AS 2.5**	91 (±1.58)	93.6 (±2.07)	96.6 (±2.3)
**AS 1.25**	73.4 (±2.07)	75.2 (±1.92)	74 (±1.58)
**AS 0.625**	51 (±1.58)	48.8 (±3.03)	51.2 (±3.49)
**AS 0.312**	25.2 (±2.59)	24 (±2.24)	24.2 (±1.92)
**AA 5**	93.2 (±2.39)	99.4 (±0.89)	99.2 (±1.30)
**AA 2.5**	87.4 (±1.67)	93 (±1.58)	94.2 (±2.39)
**AA 1.25**	72.4 (±2.07)	70.4 (±3.44)	74.4 (±2.07)
**AA 0.625**	47.8 (±1.92)	50 (±2.12)	52.6 (±2.70)
**AA 0.312**	23.6 (±2.97)	22.2 (±2.17)	22.8 (±2.77)
**CS 5**	85 (± 1.58)	90.2 (± 2.59)	91.2 (± 1.92)
**CS 2.5**	72 (± 1.58)	74.4 (± 4.83)	84. 4 (± 3.05)
**CS 1.25**	53.6 (± 2.07)	51.6 (± 1.67)	57.2 (± 3.19)
**CS 0.625**	29 (± 2.12)	31.8 (± 1.92)	34.2 (± 1.79)
**CS 0.312**	17 (± 1.58)	14.8 (± 1.79)	16.8 (± 1.48)
**CP 5**	95.4 (±1.14)	99.8 (± 0.45)	98.2 (± 2.49)
**CP 2.5**	88.4 (±1.95)	88.6 (± 1.52)	91.6 (± 2.3)
**CP 1.25**	71.4 (±2.07)	72 (± 1.58)	72.6 (± 2.3)
**CP 0.625**	45.4 (± 2.07)	44.2 (± 2.17)	44.6 (± 2.08)
**CP 0.312**	23.8 (± 1.48)	22.6 (± 1.82)	22 (± 1.58)
**SH 5**	91 (± 1.58)	95.6 (± 2.07)	96 (± 2.55)
**SH 2.5**	87.6 (± 1.67)	90.6 (± 1.14)	89.8 (± 2.17)
**SH 1.25**	69.6 (± 2.07)	72.4 (± 2.41)	71 (± 2.92)
**SH 0.625**	47.6 (± 2.3)	46.6 (± 1.67)	49.4 (± 1.67)
**SH 0.312**	22.4 (± 1.14)	21.8 (± 1.64)	23.2 (± 1.30)
**CO 5**	86.2 (± 1.92)	91 (± 1.87)	91.4 (± 1.67)
**CO 2.5**	71.6 (± 1.14)	74.4 (± 1.14)	81.8 (±2.17)
**CO 1.25**	52.8 (± 1.79)	50.8 (± 1.30)	56.2 (± 1.64)
**CO 0.625**	29.6 (± 1.82)	32.4 (± 2.30)	35 (± 2.24)
**CO 0.312**	18 (± 1.59)	15.6 (± 1.67)	17.4 (± 1.14)

SDM- standard deviation of the mean; E (ethanol), PD (potassium dichromate), AS (*A. sativum* L.), AA (*A. absinthium* L.), CP (*C. pepo* L.), CS (*C. sativum* L.), SH (*S. hortensis* L.), CO (*C. officinalis* L.).

Table S2. The mean percentage of embryogenesis inhibition (±SDM) from the experimental groups and controls, at 2, 14 and 21 days.

Time	DW	E 35	AS 5	AA 5	CS 5	CP 5	SH 5	CO 5	F	P
**2**	4.2±1.92^{d}	84 ± 1.12^{c}	94.6 ±1.2^{ab}	93.2 ±1.2^{ab}	85 ± 0.71^{c}	95.4±0.47^{a}	91 ± 0.6^{b}	86.2±0.68^{c}	362.67	0
**14**	2.8±1.79^{d}	89.8±1.36^{c}	99.8±0.26^{a}	99.4±0.44^{a}	90.2 ±1.16^{c}	99.8±0.18^{a}	95.6±0.78^{b}	91 ± 0.66^{c}	418.56	0
**21**	3.4 ± 2.7^{c}	90.2±1.36^{b}	99.6±0.51^{a}	99.2±0.65^{a}	91.2±0.86^{b}	98.2±1.02^{a}	96 ± 0.96^{a}	91.4±0.59^{b}	299.37	0
**Time**	**DW**	**E 17.5**	**AS 2.5**	**AA 2.5**	**CS 2.5**	**CP 2.5**	**SH 2.5**	**CO 2.5**	**F**	**P**
**2**	4.2±1.92^{c}	70.2±1.69^{b}	91 ± 0.91^{a}	87.4±0.84^{a}	72 ± 0.71^{b}	88.4 ± 0.8^{a}	87.6 ±0.63^{a}	71.6 ± 0.4^{b}	443.58	0
**14**	2.8±1.79^{c}	74.2±1.96^{b}	93.6 ± 1.2^{a}	93 ± 0.79^{a}	74.4 ±2.16^{b}	88.6±0.62^{a}	90.6 ±0.43^{a}	74.4 ± 0.4^{b}	242.09	0
**21**	3.4 ± 2.7^{d}	78.2±1.05^{c}	96.6±1.33^{a}	94.2 ±1.2^{ab}	84.4 ±1.36^{c}	91.6±0.94^{ab}	89.8±0.82^{b}	81.8±0.77^{c}	206.42	0
**Time**	**DW**	**E 8.75**	**AS 1.25**	**AA 1.25**	**CS 1.25**	**CP 1.25**	**SH 1.25**	**CO 1.25**	**F**	**P**
**2**	4.2±1.92^{c}	52 ± 1.8^{b}	73.4 ± 1.2^{a}	72.4±1.03^{a}	53.6±0.93^{b}	71.4 ±0.85^{a}	69.6 ±0.78^{a}	52.8±0.63^{b}	220.75	0
**14**	2.8±1.79^{c}	50.2 ± 2.2^{b}	75.2 ±1.11^{a}	70.4±1.72^{a}	51.6±0.75^{b}	72 ± 0.65^{a}	72.4 ±0.91^{a}	50.8±0.46^{b}	243.53	0
**21**	3.4 ± 2.7^{c}	56 ± 1.94^{b}	74 ± 0.91^{a}	74.4±1.03^{a}	57.2±1.43^{b}	72.6 ±0.94^{a}	71 ± 1.1^{a}	56.2±0.58^{b}	148.62	0
**Time**	**DW**	**E 4.375**	**AS 0.625**	**AA 0.625**	**CS 0.625**	**CP 0.625**	**SH 0.625**	**CO 0.625**	**F**	**P**
**2**	4.2 ±1.92^{d}	28.6 ±1.07^{c}	51 ± 0.91^{a}	47.8±0.96^{ab}	29 ± 0.95^{c}	45.4 ±0.85^{b}	47.6±0.87^{ab}	29.6 ± 0.64^{c}	146.42	0
**14**	2.8±1.79^{d}	31.2±1.36^{c}	48.8±1.75^{ab}	50 ± 1.06^{a}	31.8 ±0.86^{c}	44.2 ±0.89^{b}	46.6±0.63^{ab}	32.4 ± 0.81^{c}	105.66	0
**21**	3.4 ± 2.7^{d}	33.2±1.61^{c}	51.2 ±2.01^{a}	52.6±1.35^{a}	34.2 ± 0.8^{c}	44.6 ±0.85^{b}	49.4 ±0.63^{a}	35 ± 0.79^{c}	100.57	0
**Time**	**DW**	**E 2.187**	**AS 0.312**	**AA 0.312**	**CS 0.312**	**CP 0.312**	**SH 0.312**	**CO 0.312**	**F**	**P**
**2**	4.2±1.92^{c}	15.2±1.36^{b}	25.2 ± 1.5^{a}	23.6±1.49^{a}	17 ± 0.71^{b}	23.8 ± 0.6^{a}	22.4 ±0.43^{a}	18 ± 0.56^{b}	27.90	0
**14**	2.8±1.79^{c}	14 ± 1.12^{b}	24 ± 1.29^{a}	22.2±1.08^{a}	14.8 ± 0.8^{b}	22.6 ±0.74^{a}	21.8 ±0.62^{a}	15.6±0.59^{b}	32.32	0
**21**	3.4 ± 2.7^{c}	16.4±1.07^{b}	24.2 ±1.11^{a}	22.8±1.38^{a}	16.8±0.66^{b}	22 ± 0.65^{a}	23.2 ±0.49^{a}	17.4 ± 0.4^{b}	32.06	0

SDM-standard deviation of mean; F-statistical value (degree of freedom); p-value < 0.05 was considered significant; E (ethanol), PD (potassium dichromate), AS (*A. sativum* L.), AA (*A. absinthium* L.), CP (*C. pepo* L.), CS (*C. sativum* L.), SH (*S. hortensis* L.), CO (*C. officinalis* L.); Values with no common superscript in a column within an experiment were significantly different ($p < 0.05$). ^{a}-These values are not statistically significant from those with superscript $^{ab.}$ ^{b} – these values are statistically significant or different from those with superscript $^{a.}$ ^{c} – these values are statistically significant or different from those with superscript $^{a,\ b,\ ab.}$ ^{d} – these values are statistically significant or different from those with superscript$^{a,\ b,\ ab,\ c.}$

Table S3. The mean percentage of embryogenesis inhibition (±SDM) from the experimental groups, at 2, 14 and 21 days.

Time	AS 5	AA 5	CS 5	CP 5	SH 5	CO 5	F	P
2	10.6 ± 2.3^{ab}	9.2 ± 2.57^{ab}	1 ± 0.33^{c}	11.4 ± 1.15^{a}	7 ± 0.84^{b}	2.2 ± 0.63^{c}	17.50	0
14	10 ± 9.4^{ab}	9.6 ± 1.28^{ab}	0.4 ± 0.16^{c}	10 ± 0.79^{a}	5.8 ± 1.4^{b}	1.2 ± 0.3^{c}	18.30	0
21	9.4 ± 1.81^{a}	9 ± 0.86^{a}	1 ± 0.24^{b}	8 ± 1.17^{a}	5.8 ± 1.24^{a}	1.2 ± 0.34^{b}	12.71	1e-04
**Time**	**AS 2.5**	**AA 2.5**	**CS 2.5**	**CP 2.5**	**SH 2.5**	**CO 2.5**	**F**	**P**
2	20.8 ± 0.83^{a}	17.2 ± 2.57^{a}	1.8 ± 0.73^{b}	18.2 ± 1.94^{a}	17.4 ± 1.4^{a}	1.4 ± 0.4^{b}	39.46	0
14	19.4 ± 4.61^{a}	18.8 ± 1.05^{a}	0.2 ± 0.08^{b}	14.4 ± 1.35^{a}	16.4 ± 1.16^{a}	0.2 ± 0.04^{b}	81.09	0
21	18.4 ± 2.88^{a}	16 ± 2.23^{a}	6.2 ± 1.32^{bc}	13.4 ± 1.44^{a}	11.6 ± 0.87^{ab}	3.6 ± 1.04^{c}	15.78	0
**Time**	**AS 1.25**	**AA 1.25**	**CS 1.25**	**CP 1.25**	**SH 1.25**	**CO 1.25**	**F**	**P**
2	21.4 ± 3.28^{a}	20.4 ± 1.37^{a}	1.6 ± 0.65^{b}	19.4 ± 1.99^{a}	17.6 ± 1.57^{a}	0.8 ± 0.23^{b}	48.03	0
14	25 ± 3.08^{a}	20.2 ± 3.54^{a}	1.4 ± 0.57^{b}	21.8 ± 0.9^{a}	22.2 ± 2.03^{a}	0.6 ± 0.17^{b}	57.68	0
21	18 ± 2^{a}	18.4 ± 3.1^{a}	1.2 ± 0.48^{b}	16.6 ± 0.98^{a}	15 ± 1.49^{a}	0.2 ± 0.04^{b}	51.33	0
**Time**	**AS 0.625**	**AA 0.625**	**CS 0.625**	**CP 0.625**	**SH 0.625**	**CO 0.625**	**F**	**P**
2	22.4 ± 2.4^{a}	19.2 ± 2.36^{a}	0.4 ± 0.16^{b}	16.8 ± 1.08^{a}	19 ± 0.71^{a}	1 ± 0.29^{b}	131.62	0
14	17.6 ± 3.04^{ab}	18.8 ± 2.57^{a}	0.6 ± 0.18^{c}	13 ± 1.7^{b}	15.4 ± 0.68^{ab}	1.2 ± 0.27^{c}	56.32	0
21	18 ± 4.35^{a}	19.4 ± 2.58^{a}	1 ± 0.42^{b}	11.4 ± 2.02^{a}	16.2 ± 1.62^{a}	1.8 ± 0.52^{b}	26.145	0
**Time**	**AS 0.312**	**AA 0.312**	**CS 0.312**	**CP 0.312**	**SH 0.312**	**CO 0.312**	**F**	**P**
2	10 ± 4.41^{ab}	8.4 ± 3.02^{ab}	1.8 ± 0.73^{c}	8.6 ± 0.67^{a}	7.2 ± 1.06^{a}	2.8 ± 0.8^{bc}	7.577	0.001
14	10 ± 2.34^{a}	8.2 ± 2.2^{a}	0.8 ± 0.32^{b}	8.6 ± 1.4^{a}	7.8 ± 1.11^{a}	1.6 ± 0.46^{b}	11.90	1e-04
21	7.8 ± 1.3^{a}	4.4 ± 2.38^{ab}	0.4 ± 0.12^{b}	5.6 ± 1.03^{a}	6.8 ± 0.49^{a}	1 ± 0.19^{b}	15.61	0

SDM-standard deviation of mean; F-statistical value (degree of freedom); p-value < 0.05 was considered significant; AS (*A. sativum* L.), AA (*A. absinthium* L.), CP (*C. pepo* L.), CS (*C. sativum* L.), SH (*S. hortensis* L.), CO (*C. officinalis* L.); Values with no common superscript in a column within an experiment were significantly different ($p < 0.05$). ^{a}-These values are not statistically significant from those with superscript $^{a,\ ab.}$ ^{b}– these values are statistically significant or different from those with superscript $^{a,\ ab,\ c.}$ ^{c}– these values are statistically significant or different from those with superscript $^{a,\ ab,\ bc.}$

Table S4. Ontologies/pathogens, diseases, medicinal plants and chemical compounds used in the experiment.

Traits	**ATOL***, **AHOL****, **OPL*****, **IPNI******, and **ChEBI******* References
**Parasite load traits**	
Parasite Eggs	Egg Stage
Parasite Larvae	Larvae Stage
**Parasite used**	
Ascaris suum	AHOL_0004179
**Disease description**	
Ascaridiosis	AHOL_0005382
**Medicinal plants used**	
Allium sativum L.	528796-1
Artemisia absinthium L.	300106-2
Coriandrum sativum L.	840760-1
Cucurbita pepo L.	292416-1
Calendula officinalis L.	187894-1
Satureja hortensis L.	457680-1
**Chemical compounds detected**	
Polyphenols (µg/mL)	26195
Tocopherols (ng/mL	135821
Sterols (µg/mL)	15889
Methoxylated flavones (ng/mL)	25241
Sesquiterpene lactones (ng/ml)	37667
Sulfoxide (µg/mL)	22093

*Traits in reference to the ontology ATOL: https://www.atol-o.com/en/atol-2/; **Traits in reference to the ontology AHOL: https://www.atol-onntologytology.com/ahol/; *** Ontology for Parasite Life cycle: http://wiki.aiisc.ai/index.php/Ontology_for_Parasite_Life_Cycle; **** International Plant Names Index-IPNI: https://www.ipni.org/p/3; ***** Chemical Entities of Biological Interest-ChEBI: https://www.ebi.ac.uk/chebi/.
